# Lateral flow assay: a promising rapid point-of-care testing tool for infections and non-communicable diseases

**DOI:** 10.2478/abm-2023-0068

**Published:** 2023-12-28

**Authors:** Kumaravel Vealan, Narcisse Joseph, Sharizah Alimat, Anandi S. Karumbati, Karuppiah Thilakavathy

**Affiliations:** Department of Biomedical Science, Faculty of Medicine and Health Sciences, Universiti Putra Malaysia, UPM Serdang 43400, Malaysia; Department of Medical Microbiology, Faculty of Medicine and Health Sciences, Universiti Putra Malaysia, UPM Serdang 43400, Malaysia; Department of Chemistry Malaysia, Ministry of Science, Technology and Innovation, Petaling Jaya 46661, Selangor, Malaysia; Centre for Chemical Biology and Therapeutics, Institute for Stem Cell Science and Regenerative Medicine, Bangalore 560065, India; Malaysian Research Institute on Ageing (MyAgeing), Universiti Putra Malaysia, UPM Serdang 43400, Selangor, Malaysia

**Keywords:** aptamer, biosensor, lateral flow assay, nucleic acid, point-of-care testin

## Abstract

The point-of-care testing (POCT) approach has established itself as having remarkable importance in diagnosing various infectious and non-communicable diseases (NCDs). The POCT approach has succeeded in meeting the current demand for having diagnostic strategies that can provide fast, sensitive, and highly accurate test results without involving complicated procedures. This has been accomplished by introducing rapid bioanalytical tools or biosensors such as lateral flow assays (LFAs). The production cost of these tools is very low, allowing developing countries with limited resources to utilize them or produce them on their own. Thus, their use has grown in various fields in recent years. More importantly, LFAs have created the possibility for a new era of incorporating nanotechnology in disease diagnosis and have already attained significant commercial success worldwide, making POCT an essential approach not just for now but also for the future. In this review, we have provided an overview of POCT and its evolution into the most promising rapid diagnostic approach. We also elaborate on LFAs with a special focus on nucleic acid LFAs.

Diagnostics inarguably hold an important contribution toward healthcare in our vastly growing planet, for example, by providing suitable and convenient care to patients, guaranteeing safe blood banking, and giving vital surveillance information to both emergency public health interventions and long-haul general health strategies [1]. Notwithstanding the importance, however, they will in general get less consideration than research efforts concentrated on novel therapeutics or preventive methodologies due to the requirement of modern laboratory infrastructure, skilled personnel, and financial support [1]. Lately, the outbreak of severe acute respiratory syndrome coronavirus 2 (SARS-CoV-2) has caused a global-level alert on the importance of the existence and improvisation of suitable diagnostic approaches that can help detect the cause of the disease [2].

Diagnostic approaches can be divided into conventional, modern, and rapid diagnostic methods [3]. A simple overview of different diagnostics methods is shown in **Figure 1**. Conventional diagnostic methods are the earliest detection strategies when modern science was first embraced and require a microscope as primary detection tool to identify pathogens [3]. The identification of infectious agents is done by observing the phenotypic characteristics and by carrying out biochemical analysis [4]. Although laborious these methods are still used in many laboratories even today due to the reliability and defining costs [5].

**Figure 1.**
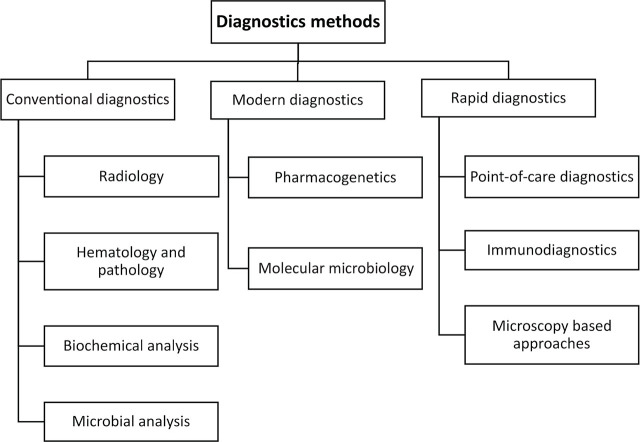
Types of diagnostic methods.

From traditional means, the diagnostic approaches have shifted to molecular and genetic studies, providing even more benefits in a brief conceivable time [3]. According to Leland and Ginocchio (2007) [6], technological advances extending from the development of monoclonal antibodies to the presentation of molecular diagnostics have given incredible assets to use in attempting to recognize the cause of viral infections. These types of diagnostic approaches have remarkable superiority in circumstances where microorganisms cannot be recognized by microscopic techniques or where microbes cannot develop outside their hosts, eventually becoming the preferred choice to recognize certain pathogens that cannot be identified by phenotype-based methods [7]. This was only possible by gene discovery, using high-throughput techniques such as next-generation sequencing (NGS) [8, 9]. Techniques such as high-performance liquid chromatography (HPLC), gas chromatography (GC), and mass spectrometry were eventually used for the separation and quantitative analysis of pharmaceutical and biological products [10, 11].

Recently, rapid detection systems, especially point-of-care testing (POCT), are being widely used compared to standard laboratory testing that highly relies on the isolation of the bacterial or viral pathogen and performing highly incomprehensive complex molecular tests [10, 11]. The diagnosis results can be obtained more rapidly by using POCT, as these advances have quickened the detection of pathogenicity at the molecular level and can recognize a large range of disease agents [1]. Thus, POCT is essential to providing rapid test results in different clinical settings to diagnose diseases [12]. They have been actively used in the emergency department (ED) where they help to improve patient care and prevent infection spread [13]. The benefits seen with the use of POCT have led to the production of a greater variety of biosensors and bioanalytical assays that can perform on-the-spot diagnoses. At the same time, the growth of POCT devices has also concurrently helped to magnify the scope of personalized medicine among the public [14]. Lately, nanobiosensors are becoming one of the most prominent diagnostic tools that use nanomaterials to provide more effective and rapid detection results.

This review briefly gives an overview of POCT and its evolution into the most-promising rapid diagnostic approach. The review also elaborates on lateral flow assays (LFAs) and nucleic acid lateral flow assays (NALFAs) and their significance in medical diagnosis and various other fields.

## Point-of-care testing

The POCT is a rapidly growing field of clinical diagnostics that is expected to be one of the primary drivers of the future in vitro diagnostic industry. It is an auxiliary, bedside, and decentralized testing, often done at the site of patient care [7]. This technique does not require a specific premise to carry out the tests as they can be done at primary care settings, for instance, at doctors’ workplaces, emergency units, intensive care, and other medical clinic units, operation rooms, schools, working environments, homes, and military operation areas [15]. Glucose, cardiovascular, and pregnancy tests are the main aspects that govern the vast majority of the point-of-care (POC) diagnoses in the current medical line globally [3].

Even though POCT had a slow reception when first introduced in the 1990s, the use of POCT later expanded massively due to the rise in consumers’ need for self-testing and improvement in medical technology. Today, various POCT devices and instruments are available in the market that can serve in various fields [7, 16]. They have become an essential detection strategy in different clinical settings to diagnose diseases depending on their properties and ability to recognize many types of analytes such as metabolites, proteins, and microorganisms [15]. Different properties of various POCT devices are explained in **Table 1**.

**Table 1.** Different properties of various POCT devices

**Types**	**Properties**	**References**
Qualitative strip–based handheld POCT devices	Immunochromatographic reaction Signaling depends on the simple visualization of olorimetric changes. Portable Test usually done at the site of patients’ care. E.g.: LFDs, ICSs, and multiplex LFDs	[17, 18]
Quantitative strip or chip–based with unit use analyzers	Used for quantitative analysis A reader is used to measure the reaction onto the strip. Allows to measure the INR levels of certain metabolites or proteins in our body. Test strips are for single use and disposed after the data collection. Common for blood sugar and thrombin analysis	
Bench top POCT analyzers	Requires sample containers, labelling and needed to be carried out in clinical settings. Can be set up at different POC locations. Miniaturized and increased computer processing. Used for spectrophotometric enzyme-substrate measurement, hemagglutination, immunoassay, and blood gas analysis. E.g.: agglutination assays, immunofiltration assays	
Molecular biology–based/nucleic acid POCT devices	Used for detection of pathogenic diseases. Rapidly quantifies nucleic acid from numerous infectious agents. Incorporated molecular biology techniques such as isothermal amplification reaction	

ICS, immunochromatographic strips; INR, international normalized ratio; LFDs, lateral flow devices; POC, point-of-care; POCT, point-of-care testing.

The POC-geospatial concepts are widely applied by many countries across the globe as a precaution against disaster, for potential risk awareness and management, and as communal health adversaries such as outbreaks. Integration of both the concepts helps respond quickly and control the spread of deadly outbreaks such as Ebola, Middle East respiratory syndrome-based coronavirus, and malaria. Many healthcare systems based on this concept are being employed in works to be implemented in countries with a high-risk potential of outbreaks. Termed as geospatial-POC preparedness, the ideology of preparing a mobile healthcare facility with improved and easy-to-handle diagnostic tests can improve the management of world countries with respect to outbreaks and border security [19].

The POCT tools are highly dynamic due to their advantages in diagnosing a wide range of infectious and non-communicable diseases (NCDs). This is because various types of samples can be used as the target material for conducting detection and analysis. Interestingly, several POC tests have been developed, focusing on the principal infectious disease-causing microbes, such as malaria parasites, human immunodeficiency virus (HIV), human papillomavirus (HPV), dengue, Ebola, Zika viruses, and *Mycobacterium tuberculosis* (TB) bacteria [20]. For instance, rapid diagnostic test lateral flow strips capable of detecting malaria parasite proteins have been used for the early diagnosis of malaria in tropical areas [21], enabling prompt artemisinin-combined therapy (ACT) [22]. The development of a POCT tool that can quantify CD4+ T-lymphocytes and HIV viral load for HIV detection [23], POC test platforms that can replace visual inspection with acetic acid (VIA) for HPV diagnosis [24], and the formulation of the ReEBOV antigen rapid test kit for the rapid detection of the Ebola virus in East African countries [25] provides additional supporting evidence for the use of POCT in the context of infectious diseases.

At the same time, the increase in morbidity and mortality rates in recent times due to alarming levels of NCDs such as diabetes, stroke, cancer, type-2 diabetes, and Alzheimer's disease has exponentially increased the demand for POCT applications for diagnosing these diseases [26]. The diagnosis of NCDs, which previously heavily relied upon laborious biochemical measurements, is now being replaced by simpler detection tools without compromising on detection sensitivity and specificity [27]. Recent research has reported the use of POCT to screen for individuals suffering from hypertension and obesity, improving the potential to manage these conditions while raising awareness about NCDs [28]. The diagnostic strategies for cancer have also changed their trajectory toward POCT, which previously relied upon conventional biological tests that may take up to few weeks to complete. The immunoassay-based POCT facilitates the early detection of several cancer types such as pancreatic cancer or prostate cancer, only requiring less volume of body fluid or blood sample [29]. At the same time, the use of POCT also helps in the early diagnosis of diabetes by analyzing HbA1c [30], further cementing the improved role of POCT in noninfectious diseases.

Given the current epidemic situation caused by SARS-CoV-2, POCT has emerged victorious among the other conventional diagnostic principles such as reverse transcriptase-polymerase chain reaction (RT-PCR) [31]. This is due to the ability of POCT to offer rapid diagnosis and accurate and timely detection, and the tests can be carried out at the user's end, thus exponentially increasing the rate of diagnosis in a short time [32]. The rapid test kits for SARS-CoV-2 detection are readily available in pharmacies and healthcare facilities, and the public can get them at a very low cost to perform their detection test.

## Designing and optimization of POCT

The POCT devices are mainly designed based on membrane-based microfluidic technology. This is because other materials such as glass, silicon, or polymers would require a complex designing mechanism involving extensive processes and additional instruments. This would increase the overall manufacturing expenditure of the devices, making them not compatible with POCT. Papers, on the other hand, are easy to fabricate and do not require specific cutting mechanisms and are regularly used for most of the POCT devices design. Thus, these devices are also known as paper-based analytical devices [33].

Cellulose and nitrocellulose are the two main kinds of membranes used for POCT device development and manufacturing [34]. Cellulose is a linear macromolecule made up of an array of glucose units. They are organic solvents and insoluble in water. Filter paper and chromatography paper are examples of cellulose-based paper materials and are often used for dipstick assays and microfluidic devices (μPADs) [17]. On the other hand, a partial nitration process is carried out to produce nitrocellulose, where this process converts the hydrophilic property of cellulose into a hydrophobic one. Lateral flow devices (LFDs) are mostly designed using this material [33]. Both cellulose and nitrocellulose are porous materials, and nitration improves the porosity of the materials, granting a slight advantage to nitrocellulose over cellulose. The porosity shows the wetness of the material and determines the flow of liquid on the membrane. Moreover, the surface chemistry of these membranes is also given special attention due to its influence on particle immobilization, adsorption, and colorimetric changes during the assay process [34].

POCT devices usually come with a simple design, where most of them are available in pure disposable form. They are formatted for single-use and can detect antigens or antibodies from a wide array of specimens. Lateral flow and immunochromatographic strips (ICS) are examples of such devices. Due to their simple nature, the production cost is very low, and they are inexpensive compared to permanently integrated device strategies such as polymerase chain reaction (PCR). Moreover, these devices are affordable in most of the developing countries with resource-limited settings [35]. In some cases, the disposables are integrated with a reader to get the reading of the analyte detected, such as found in a blood glucose analyzer [35, 36].

On the other hand, these testing devices are designed to be lightweight, portable, and user friendly such that they can perform the complex tests at the site where they are most needed [7]. They can provide detection results instantly due to limited or no sample processing steps that accelerate the testing process. Hence, raw samples such as urine or blood can be used as primary samples for analyte detection [15].

## POCT detection technologies

Before POCT, detection strategies heavily relied on complex techniques such as microtiter plate reader devices. An electrical power source was needed to operate the devices, making them non-portable and the tests needed to be conducted in laboratories. However, with the introduction of POCT that primarily uses immunoassay technologies, detection can be easily done with maximum sensitivity. The use of immunoassay techniques also helps in coping with the need for rapid turnaround times of the test. There are three assay techniques such as agglutination, immunofiltration, and immunochromatographic assays [7].

### Agglutination assays

The agglutination assays involve the aggregation of erythrocytes with a small portion of latex materials. A small volume of sample will be mixed with particles such as latex bead coated with analyte-specific antigens or -antibodies, and the aggregation can be analyzed visually [15]. Only serum, plasma, or urine can be used as samples for these assay formats [12]. Agglutination assays can be performed in a single step, following the homogenous assay principle and the results are obtained in minutes. These assays necessitate high analyte concentration for better visibility and the results are semiquantitative at best [7]. In 2003, the first latex agglutination test was carried out by Pro-Lab Diagnostics, South Wirral, the UK, for the sero-grouping of ß-hemolytic streptococci [7]. Very recently, a multidisciplinary group of researchers has produced a simple hemagglutination test (HAT) for the detection of the antibodies to the receptor-binding domain (RBD) of the SARS-CoV-2 spike protein. The SARS-CoV-2 RBD is linked to the red blood cells by targeting the single-domain antibody IH4 via a linker, forming an IH4–RBD complex. This HAT strategy helped to channel a more affordable and easy-to-use serological technique for the detection of antibodies to SARS-CoV-2 that can be used to neutralize the virus via suitable vaccine production [37].

### Immunofiltration assays

In immunofiltration assays, the sample together with various reagents and wash solutions is filtered sequentially through a porous membrane. The membrane contains spots or lines of immobilized antibodies [7]. The assays are performed with multiple manual tests, including the application of samples and reagents at the right proportion. Despite following the heterogeneous assay principle, the results are obtained in a very short time due to quickened interaction of analytes with immobilized antibodies via filtration and effective washing steps [15]. Colored conjugates are often used to analyze the different analytes present in the sample. The sensitivity of these assays is very low, where the results will be yielded at the nanomolar range and are now very rarely used for detection [7]. At the end of 2004, Axis Shield Diagnostics Ltd, an organization based in Scotland and pioneering in manufacturing innovative in vitro diagnostics tests developed a simple immunofiltration detection system to measure the level of HbA1c in red blood cells [7]. The strategy was then converted into the agglutination format due to improved sensitivity, and the agglutination can be read turbidimetrically [38]. In 2016, a research institution developed the Antibody Immuno Column for Analytical Processes (ABICAP) immunofiltration system that is primarily used to detect orthopoxviruses that causes severe diseases to humans. The assay results were obtained within 45 min, much faster than immunosorbent assays, and offered great sensitivity than a conventional LFA for the diagnosis of this virus [39].

### Immunochromatographic assays

Immunochromatographic assays are based on the movement of targeted analytes and antibody conjugates via the drive of capillary force and produce colored lines as signals upon specific capture-antibody or control-antibody interaction at their specific zones. Compared to agglutination and immunofiltration assays, manual immunochromatographic principle–based POC immunoassays are having a storm at the international market, making them the most popular POCT immunoassay used for diagnosis purposes. They in general have higher sensitivity and are easy to handle compared to the other assay formats mentioned earlier [15]. The LFDs and ICS tests use this assay principle in nature where the colored capture particles with significant optical properties produce bright visible lines, indicating the outcome of the assay. Gonorrhea, chlamydia, and syphilis are a few examples of the very important diseases that were first to employ the ICS tests for detection, and this later helped to control the severity of the disease [40].

## Nanobiosensors-integrated POCT

A biosensor is designed on the principle of analyzing a biomolecule quantitatively and converting the result into a visible signal [41]. With the current trend of exploiting the science of nanotechnology becoming widespread, the adaptation of this technology in the POCT approach enabled the development of nanobiosensors as advanced bioanalytical devices [38, 39].

Nanobionsensors are no different from any of the other kinds of biosensors or bioanalytical devices available. In fact, they are used for the same purpose, which is to quantify the biochemical or any biological event by the means of electrical, optical, or magnetic technology. However, unlike other conventional biosensors, nanobiosensors use a compact probe that is derived from various nanomaterials [40, 41]. The nanomaterials used can show their novel characteristics better compared to the original materials that do not have nanoscale features. Several nanomaterials have been used for the biosensing devices such as nanotubes, nanorods, nanoparticles, and nano-thin films [42].

Nanobiosensors overcome most of the limitations identified in conventional biosensors that became a barrier to expanding their use in various fields. This is because the nanoparticle size and shape can be optimized during synthesis to reach the maximum electrochemical active sites [43]. The nanomaterials are also designed to provide high sensitivity for detection; hence, they can locate the analytes from the sample and eventually provide outcomes with maximum precision [43]. In cases where noble metal nanoparticles such as colloidal gold nanoparticles (AuNPs) are used, optical sensing can be vastly improved by enhancing the surface plasmon resonance (SPR). Reducing the SPR intensity of the nanoparticles allows them to collect more light from the surroundings due to their optical sensitivity, and signals with great intensity can be produced [44].

As of today, several forms of nanobiosensors are available and used in various fields such as biomedicine, optics, catalysis, and electronics. However, affinity-based nanobiosensors that are available in the simplest form possible remain atop of the others. In other words, LFAs are the most-preferred nanobiosensing tools, operating based on the affinity of the biorecognition molecule to the sample and forming a complex [45].

### Lateral flow assays (LFAs)

The LFAs are the paper-based POCT biosensors that are primarily used for the detection and quantification of a specific target from a complex mixture [46]. They come as preassembled strips of a transporter material encasing dry reagents that are activated by applying the sample of interest in liquid form and the eventual result can be observed from 5 min to 30 min [47]. LFAs are accountable for single use at the POC [48] such as when used to determine pregnancy, the collapse of inner organs, the pathogenic effect of specific microorganisms including biowarfare agents, a toxic mixture in consumables, feed and environment, and illicit drug abuse [47].

A typical LFA consists of an application pad, conjugate pad, nitrocellulose membrane, and adsorption pad mounted on plastic backing [49]. The pads and membrane are generally porous. The pores in the sample pad can be distributed symmetrically or asymmetrically and they help to filter any coarse materials from the sample before reaching subsequent sections of the instrument [48]. The whole assay works depending on the wettability and capillary flow action; hence, the pore walls need to be wetted by the sample liquid. The sample is dropped onto the sample pad after mixing with viscosity enhancers or buffer salts that enhance the sample viscosity and improve the overall flow rate [50]. Cellulose filters and cross-linked silica are two common materials used to manufacture the pads, and the selection of the material heavily relies on the objectives of the test and characteristics of the sample [45, 51].

In the conjugate pad, labelled conjugate elements have been applied and dried during the manufacturing process. When the buffer-diluted sample is applied to the sample pad, it migrates to the conjugate pad via capillary action and dissolves the conjugate element in the liquid. The interaction between the sample and the conjugate element results in the sample-label tagged conjugate complex formation, which is imminent for the final reaction at the membrane. By filling the pores in the pad, the newly formed complex can migrate to the reaction membrane [48].

**Figure 2.**
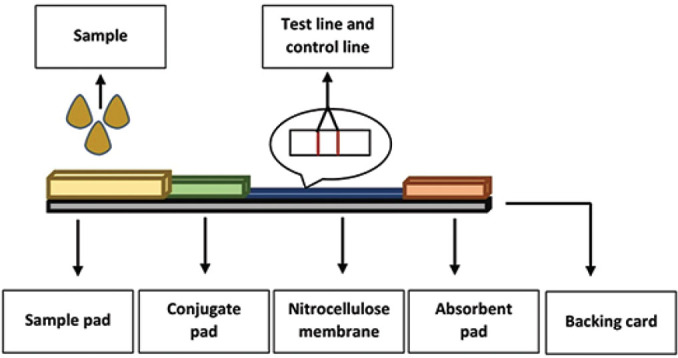
Components of lateral flow assays.

Reaction membrane is an important part of the LFA instrument. Very often, the reaction membrane is designed using nitrocellulose due to its porosity. Nylon, polyethylene, and fused silica are a few new materials that have been used to design in some of the recent research. The affinity of the sample, such as a protein, with the porous structure is very important for the interaction with the capture probes available in the test line and control line [52]. The test line probe and control line probe are immobilized onto the membrane to be recognized by the complex. The colorimetric changes at the test line depending on the presence and absence of the analyte of interest determine the reaction, while a signal at the control line validates the assay [46]. The components of the LFA strip are shown in **Figure 2**.

The sample pad is the proximal end of the strip where the sample is placed for a test run. This is the area where the sample is also treated. The treated sample will flow through the strip to the conjugate pad and form a conjugate with label-tagged conjugate probes. Normally, conjugate particles that are used in LFA have unique optical, electronic, and/or structural properties such as AuNPs and up-conversion nanoparticles. The analyte then migrates to the reaction matrix membrane or more commonly known as nitrocellulose membrane where a control line and a test line are fabricated by depositing control probes and capture probes. Nitrocellulose membrane is the main material in LFAs as it grants a stage for reaction and detection during the assay. At the end of the strip, the wick or absorbent pad collects the excess reagent from the membrane.

In the past decade, the use of LFAs has been expanded to various fields, which simultaneously skyrocketed the need for them, especially where rapid tests have become prevalent. This is because these diagnostic devices can emulate the criteria gazetted by the World Health Organization (WHO) that specifies the requirement for an ideal POC device. The criteria are known as the “ASSURED test” where the diagnostic tools should be affordable, sensitive and specific, user-friendly, rapid and robust, equipment-free, and deliverable [53]. LFAs come with a simple designated structure, making them portable and equipment-free. The manufacturing cost is very low compared to other diagnostic tools; hence, LFAs are available in the market at an affordable range. The assays can also be performed at the patients’ site of care by anyone without needing any complex procedure or skilled personnel. Thus, LFAs are widely regarded as ideal POC devices and are preferred the most by consumers and regulatory authorities [46]. The different sensing modalities and advantages and disadvantages of LFAs have been explained in **Table 2**.

**Table 2.** Sensing modality, features, advantages, and disadvantages of various LFAs

**Sensing modality**	**Features**	**Advantages**	**Disadvantages**	**References**
Optical	Colorimetric	Colorimetric changes help to make a visual observation for result interpretation	Sensitivity issues that lead to result inaccuracy	[53]
	Fluorescence	Provides results with higher signal to noise ratio; higher sensitivity	Fluorescent labels are not visible at low concentration; requires external reader for specific analysis	[54]
	SERS	High sensitivity with very low detection limit; can be developed into ultiplex LFA for a wide range of detection	Expensive	[55]
	Chemiluminescence	High sensitivity when used together with metal nanoparticles; lower LOD; highly stable for immobilization	Requires addition of enzymatic substrate; low shelf-life	[56]

Thermal	Thermal imaging	Improved LOD and high sensitivity; no photo bleaching or photo instability	The infrared image sensors are massive; expensive	[57]
	Laser speckle imaging	Uses improved depth resolution reader that improves sensitivity	Expensive and not suitable at resource-limited settings	[58]
	PA imaging	PA imaging helps sample penetration deeper that improves the sensitivity of detection	Strips need to be dried before analysis since acoustic waves travelling through water-interface is a challenge	[44, 59]

Magnetic	MPQ	Low detection limit and highly sensitive	Not suitable for multiplex detection when using high affinity variants	[52, 60]

Electrochemical	Amperometry, cyclic voltametric, impedimetric	The ratio of applied voltage can be adjusted to increase the sensitivity of the detection; nanoparticle integration to improve LOD	Requires expensive reagents and multistep procedure	[44, 61]

LFA, lateral flow assay; LOD, limit of detection; MPQ, magnetic particles quantification; PA, photoacoustic; SERS, surface enhanced Raman scattering.

### LFA detection system

For LFAs to function effectively, the choice of a label detection marker possesses an important role. An ideal detection marker is expected to be nontoxic, simple, and have high detection sensitivity, does not disrupt assay progress, and have zero effect on the long-term stability of the conjugate [59]. Hence, nanoparticles were employed as the suitable detection markers to be incorporated with LFAs. Various types of nanoparticles were used such as colloidal gold, carbon, selenium, silver, organic fluorophores, and colored latex beads that enabled a wide range of possibilities for the introduction of LFAs as nanobiosensors [46].

Even though multiple labeling-detection systems are being introduced from time to time, colloidal gold remains to be the gold standard among researchers and is highly preferred compared to others. Colloidal gold is chemically inert, which gives very perfect spherical particles. The spherical structure is associated with the ability to absorb and reflect light depending on the depth of electromagnetic field produced around the nanoparticles [49]. Hence, a bright red color can be produced as a visible signal after the assay progression is completed [49]. Compared to other materials used, colloidal gold can form a conjugate with different biomolecules effectively, enabling quick and long-lasting conjugation of antibodies, aptamers, and other targeting moieties regularly used for lateral flow tests [46].

### Lateral flow assay design

**Figure 3.**
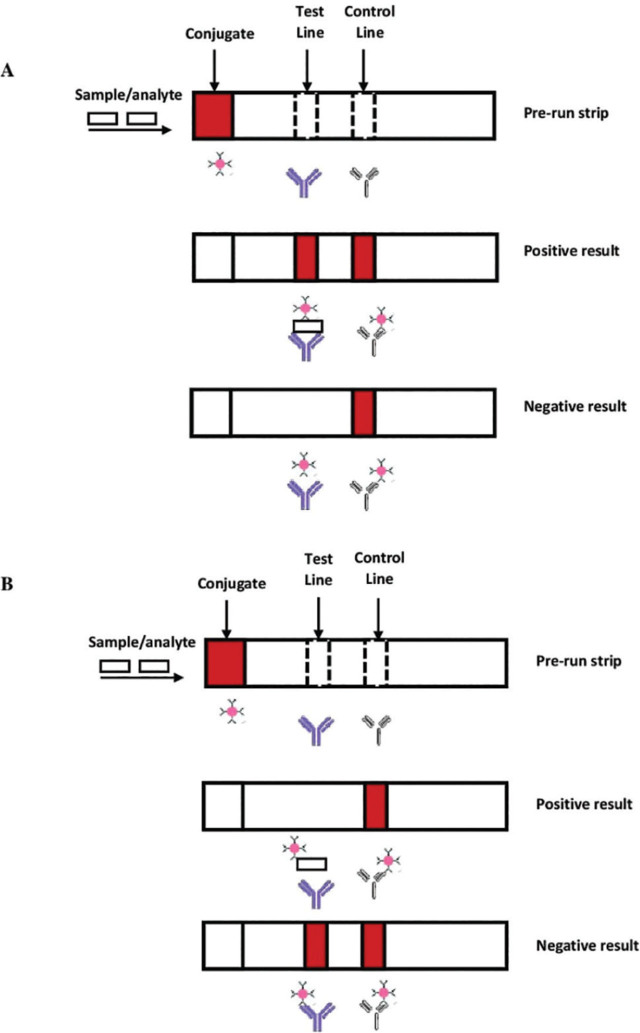
Lateral flow assay designs. **(A)** Sandwich assay or not competitive assay: A visible signal produced at both the test line and control line indicates the positive result. The analyte is present in the sample and hybridized to the capturing molecule at the test line. The absence of analyte gave a negative result where the signal was only produced at the control line. The signal at the control line is a result of the hybridization of the capturing molecule at the control line region that is complementary to the free conjugate molecules. **(B)** Competitive assay: A visible signal only at the control line gave a positive result, indicating the competition of analytes with the capturing molecule and no aggregation of conjugated particles at the test line. However, the signal at both the test line and control line gave a negative result, indicating the absence of analytes in the sample and binding of conjugated particles to the capturing molecules at the test line.

LFA protocols can be divided into two groups, i.e., competitive (inhibition) and noncompetitive (sandwich) assays [62]. The mechanisms of competitive and noncompetitive assays are shown in **Figure 3**.

In a sandwich assay, the analyte with multiple determinant sites from the sample reacts and hybridizes with the conjugate particles to produce analyte–conjugate complexes at the conjugate region. The complexes now migrate along the membrane via capillary force. Once the complexes reach the test line, they interact with the immobilized capturing molecules that are complementary to one of the determinant sites, forming a conjugate–analyte–capture molecule complex. The analyte is sandwiched between the conjugate and capture molecules. The complex results in a strong visible signal formation. Free conjugate particles move past the test line and migrate to the control line. At the control line, another capturing molecule complementary to the conjugate probe interacts and forms a signal, showing LFA validation. In the absence of an analyte, no complex is formed at the test line and no signal is produced [62].

Competitive assays are suitable for testing small analytes with single determinant sites and those that cannot bind to two antibodies simultaneously. In the presence of analytes in the sample, the analytes compete with the capturing molecules for binding sites at the test line. As a result, there is no aggregation of conjugated particles at the test line; hence, no visible signal is produced and a positive result is received. When there is an absence of analyte, capturing molecules at both the test and control lines will react with the conjugated particles and produce a visible signal [63].

## Cost-effectiveness of LFA

Besides the improved diagnosis rate, cost-effectiveness also drives the implementation of LFA-based diagnosis across various medical sectors. As discussed earlier in this review, rapid test kits for SARS-CoV-2 were not only cheaper but also easily accessible to the public, enabling instant testing and screening for the infection. This helped reduce the reliance on laboratory-based testing methods like RT-PCR, which, in addition to being laborious, incurred high costs in terms of personnel, machinery, and testing facilities [64].

Furthermore, cryptococcal antigen (CRAG)-LFA was used to screen for cryptococcal meningitis (CM) among HIV patients in Uganda. CM served as a major cause of morbidity and mortality (approximately 10%–44%) among HIV patients. The use of CRAG-LFA in this resource-limited setting maximized disease diagnosis capacity and highlighted the need for additional research methods. Importantly, the use of such POCT reduced the burden on the country to establish laboratory-oriented diagnostic facilities while maintaining cost-effectiveness in line with their health economic principles[65].

In another report, it was concluded that the use of POCT in hospital EDs significantly contributes to cost management compared to using conventional diagnostic methods, further emphasizing their importance in cost-effectiveness [66]. However, it is worth noting that the cost of POCT can fluctuate, sometimes exceeding that of traditional methods, depending on the clinical setting, target condition, and test quality [67].

## Current and future development of LFA

LFAs, which are primarily based on antigen–antibody interactions, have shifted their paradigm toward using nucleic acid sequences as the sample for the detection of amplicons with the aid of PCR [68]. This allows LFAs to be used in medical conditions that cannot be diagnosed precisely using antigens and antibodies, but oligonucleotides are required, instead. Apart from this, new labels such as quantum dots and upconverting phosphors are used to improve the sensitivity of LFAs to detect analytes even at lower concentrations [69]. Nonmetallic nanoparticles such as carbon nanotubes (CNTs) and graphene oxides are now been significantly integrated with LFAS, producing CNT-based colorimetric LFAs [46]. These LFAs have been shown to produce remarkable sensitivity as that of the AuNP-mediated assays, suggesting these nonmetallic nanoparticles can replace the metallic nanoparticles in the future. In terms of improving the detection signal, enzymes like horseradish peroxidase (HRP) are used where they help to magnify the signal intensity by helping assembly of detection particles around the sample, and the chemiluminescence signal is measured using specific tools [70]. Along with chemiluminescent detection, fluorescence-based detection strategies are also widely used in LFA technology because they have lower detection limits and help in quantitative analysis [71].

Apart from optical detection, magnetic nanoparticles (MNPs) are used as labels for detection. They employ the magnetic particles quantification (MPQ) technique to quantitatively evaluate the result obtained [53]. A stray magnetic field is generated via external magnetic forces and the intensity of the magnetic field around the MNPs is quantified using the MPQ technique [72].

At the same time, few LFA detection strategies have been discovered to circumvent sensitivity problems such as using thermal response to quantify the analyte concentration in the sample. These thermal imaging–based LFAs use infrared cameras or laser speckle to detect the temperature distribution around the LFA strip under the illumination by photothermal (PT) excitation light. This PT-based thermal imaging method helps to evaluate the improvisation in the limit of detection (LOD) and sensitivity [73].

Moreover, surface-enhanced Raman scattering (SERS) LFAs are now being widely used for the detection of the influenza virus [55]. It is a surface-sensitive–based detection where the target molecules are attached to the SERS tracers; upon reaction, a Raman scattering spectrum is observed and measured using Raman spectrometer [58]. It still employs AuNPs as reporter probes and is regarded as more sensitive than conventional LFAs and hemagglutination assays [71].

Reducing the scale of the LFAs and optimization of those devices to make them even more convenient and portable is an important goal aspired to be achieved in the future [53]. Prospects like integrating LFAs in lab-on-a-chip design are underway to extend their use for more resource-limited settings [74]. Moreover, researchers also want to introduce LFAs with micro electro-mechanical systems and improve their sensory transmissions so that devices like mobile phones can be used to operate the assay or their use can be translated even in military service [53].

On the other hand, multiplex LFAs have been introduced to carry out analysis on more than a single analyte using the same test. Due to the increasing trend in discovering new biomarkers, the conventional individual biomarkers detection using multiple LFA strips is no longer an ideal approach. It is necessary to detect multiple biomarkers at once in order to produce information that is insightful or definitive in contemporary clinical settings. Hence, researchers started normalizing using multiplex LFAs that can detect all the biomarkers using a single strip, and the result was remarkable [76]. Several test lines were designed on a single strip and targets were detected via spatial resolution. This approach is often used in the food industry [75]. Multiplex LFAs, while retaining the integrity of a single LFA system, increase the detection sensitivity and reduce the overall cost for multiple tests. This helps integrate the reader system with the multiplex LFAs that can improve the result analysis while maintaining the cost at a rate suitable for low-resource settings [77].

## Nucleic acid lateral flow assays (NALFAs)

Researchers’ interest in nucleic acid-based detection became key for the invention of various biosensors and bioanalytical devices that employ nucleic acid sequences as primary samples [78]. Known as nucleic acid biosensors (NABs), they are useful for clinical diagnostics of inherited diseases and pathogenic infections [79]. One of the most promising NABs that are currently being used regularly is NALFAs.

NALFAs are paper-based analytical devices designed based on the similar structural framework of conventional LFAs that use nucleic acid samples or modified aptamers as recognition molecules [34]. To date, they are not just used for clinical diagnosis of diseases caused by pathogenic microbes, but also expanded to various other fields of research including plant pathology, veterinary diagnostic, food safety, and environmental diagnostics [80].

The assays can be divided into two formats. The first one involves the hybridization of single-stranded amplicons (ssamplicons) to complementary probes that are immobilized onto the membrane. The second format involves specific-tagged double-stranded amplicons (ds-amplicons) coupled with an antigen–antibody reaction. This format is known as nucleic acid lateral flow immunoassay (NALFIA) and requires PCR amplification of amplicons before assay [47]. Nevertheless, both formats serve a single purpose, which is to generate rapid, effective, and highly sensitive test results.

POC devices that can detect the variations in human genetic sequences are still under development and have not been generalized like other device types [81]. Researchers came very close a few times in fabricating fully functional NALFAs that can employ nucleic acid sequences to detect the nucleotide mismatches and mutations present in the sample [82]. For example, a multidisciplinary research team of the National University of Singapore (NUS) developed enVision, a device used for screening of infectious diseases using the enzyme-assisted nanocomplexes for visual detection of nucleic acids, where this device was tested for the detection of cancers markers and human genetic markers [83]. A research team developed an amplification refractory mutation system–lateral flow assay (ARMS–LFA) to study the individual genetic variations via single nucleotide polymorphisms for detection purposes and subsequently treating the genetic disorder [81]. These researches gave promising early output but still it requires improvisation. Nevertheless, these attempts have now paved the way for advanced research in developing NALFAs for human genetic disorders diagnosis.

## Isothermal amplification–based NALFAs

PCR-based techniques were extensively used for microbial-based disease detection to understand the genetics of the microorganisms, especially, the RNA viruses [84]. Currently, SARS-CoV-2 caused by an enveloped spike-like coronavirus from the genus *Betacoronavirus* has succumbed the whole world in one of the deadliest pandemics in history, spreading to almost every country in the world and causing millions of deaths to date. This is because the genomic RNA of the viruses instantly comprehends the hosts’ cellular machinery and is translated into protein, necessitating the use of PCR technology such as reverse transcription-quantitative polymerase chain reaction (RT-qPCR) for systemic detection [85].

However, qPCR was not the ideal method for the long-term detection strategy, considering the need for highly skilled personnel, centralized medical facilities, and sophisticated sample-preparation technology with automated systems. This eventually increases the overall expenditure needed to carry out the tests and would not be suitable for countries with low medical facility settings [86].

At the same time, POCT tools such as NALFAs were slowly being introduced in most medical settings, suggesting an easy way for detection. They come with great specificity but the available POCTs could not replicate the sensitivity that the nucleic acid amplification could offer [85]. Hence, the amplification strategy was revised, and an isothermal nucleic acid amplification was discovered as a proper solution to all those issues faced earlier [87]. Isothermal nucleic acid amplification is the process of effectively accumulating nucleic acid sequences at constant temperatures [88]. This amplification technique was later combined with a lateral flow system, forming isothermal nucleic acid amplification–based NALFAs [89]. This intervention allowed researchers to progress even nearer to producing devices that satisfy the ASSURED strategy [90]. This isothermal amplification is also expanded into several different formats.

## Recombinase polymerase amplification-LFA

Recombinase polymerase amplification-LFA (RPA-LFA) is a highly sensitive nucleic acid amplification technique, employing recombinase complex from T4 bacteriophage to introduce primers to specific DNA sites and begins amplification by the strand displacing DNA polymerase in the presence of adenosine triphosphate molecules [86]. It is often regarded as one of the best alternatives to PCR and believed to have subdued imperfections found in other isothermal approaches such as nucleic acid sequence-based amplification (NASBA), strand displacement amplification, rolling circle amplification, the loop-mediated isothermal amplification (LAMP), and helicase-dependent amplification [91].

RPA-LFA eliminates the template denaturation step, which is important for the PCR technique. There is no temperature fluctuation needed as this technique can operate at a low and constant temperature ranging from 38 °C to 42 °C [92]. Recombinase polymerase amplification (RPA) only requires about 10 copies of template strands for amplification, showing its greatly improved sensitivity matching with the PCR technique [84]. In one of the recent studies, it was mentioned that RPA can still produce amplification signals notably within 10 min even at the near-detection-limit template concentrations, reassuring the capability of this amplification technique as a great alternative and to be combined with lateral flow detection [86].

The RPA-LFA combination first came into light nearly a decade ago and is used for the discovery of many microbial species such as HIV-144, Canine Visceral Leishmaniasis, *Orientia tsutsugamushi*, *Rickettsia typhi*, plasmodium, intestinal protozoa, cryptosporidiosis, yellow fever virus, *Penaeus stylirostris*, *Plasmodium falciparum*, *Entamoeba histolytica*, *Schistosoma haematobium*, little cherry virus, and plum pox virus [91].

The combination allowed to increase the LOD of many NALFA formats and eventually quickened the detection process [93]. Moreover, tailed primers are often associated with RPA, anchoring for the improvised hybridization capacity of the amplicons. The use of tailed primers also surpasses the need for hapten labeling and use of capture and reporter antibodies and terminates postamplification processing [91]. The entire assay, including amplification and detection, could be completed within 15 min through all the modifications, showing the efficiency of RPA and rapid hybridization kinetics of the tailed amplicon with the surface-immobilized probe and the reporter probe and making them an effective choice for fast, low-cost and highly sensitive NALFAs [91].

## Nucleic Acid Sequence Based Amplification (NASBA)-LFA

NASBA is a transcription-based amplification and a part of the isothermal nucleic acid amplification, exclusively designed for the detection of RNA targets [81, 83]. This technique is also known as “self-sustained sequence replication” due to its capability of generating new copies of nucleic acid template sequences with or without denaturation process, the same as RPA [94]. An abundance of antisense RNA copies are produced at the end of the DNA NASBA and is usually recognized by the hybridization of specific fluorescent molecular beacons [16].

NASBA is often associated with oligochromatographic detection using specific dipsticks and is deemed an indispensable contribution in countries with poorly equipped laboratories. The amplified product is applied and allowed to migrate on the sensitized membrane of an oligochromatographic stick. Capture and detector probes functionalized with nanoparticles then hybridized with the amplified product because of migration and produced a signal as an indicator [94].

LFA will detect the amplified RNA product quantitatively. This in turn enables researchers to analyze samples with lower concentrations because NASBA can amplify the sample and eventually maximize the LOD of LFA. On the other hand, NASBA-LFAs undergo optimization including oligonucleotide concentration, buffer composition, membrane flow rate, strip size, and nanoparticle size to keep the overall cost of the LFAs as low as possible even though amplification is required. The signal produced by the NASBA-LFAs will be analyzed based on signal-to-background ratio calculation since a stereo microscope equipped with a color camera is used for nanoparticle capture analysis. LFAs with the best resolution show they can detect NASBA products, proving NASBA enables detection of short, amplified genomic sequences through LOD improvisation [93].

Recently, new work was initiated to use the NASBA technique as a testing strategy for SARS-CoV-2 Virus (COVID-19). The project was named as Isothermal NASBA-Sequencing based hIGH-throughput Test (INSIGHT). It is a two-stage COVID-19 test strategy, combining an isothermal NASBA technique with NGS. Crude saliva is employed for isothermal NASBA reaction in the first step, and billions of copies are produced within 2 h. The amplified sequence is then subjected to the NGS stage to intensify the sensitivity and specificity of the test in a scalable manner. The amplification is monitored in real-time using molecular beacon readouts, and researchers are working on the possibility of using lateral flow assay to receive rapid test outcomes [95].

## Loop-mediated isothermal amplification (LAMP)-LFA

LAMP technology is a novel nucleic acid amplification method that works on the principle of auto-cycling strand displacement DNA synthesis [96]. This action is being carried out by Bst DNA polymerase large fragments [97]. This technology was discovered with the same motive as other isothermal amplification techniques, which is to improve the sensitivity and sensitivity of nucleic acid amplification for detection. Since its inception in the year 2000, the technique was emulated directly into a diagnostic assay for the detection of various diseases and now remains alongside RPA and NASBA as one of the isothermal amplification techniques associated with LFAs [98].

LAMP uses 4–6 primers recognizing 6–8 distinct regions of target DNA for a highly effective amplification [96]. The reaction begins with a strand-displacing polymerase, catalyzing strand invasion of the template by hairpin-forming LAMP primers. These primers are responsible for annealing and extension and are later replaced by displacement primers to initiate amplification, forming a dumbbell-like structure [99]. This structure was retained throughout the process and became the mechanics for amplification [100].

Same as RPA, LAMP uses a single and constant temperature for amplification ranging from 60 °C to 65 °C. LAMP-based assays do not require thermocyclers and tedious operational mechanisms like PCR, making them easy to perform with advanced specificity and incorporate in point-of-need diagnostic tools such as LFAs to improve the detection strategy [96].

LAMP-LFAs are appropriate diagnostic tools to be used in various fields and are commonly used in the agricultural field to understand plant virology [101]. They easily replace the gel electrophoresis procedure that is commonly required in conventional PCR techniques to see the product. Even though they both have a similar role to a certain extent, LAMP-LFAs are better than electrophoresis because they come in as predesigned simple structures and do not require multiple steps [100]. The sample loading is a lot easier compared to electrophoresis where skilled personnel is required. Furthermore, the outcome can be visualized easily via LFAs whereas electrophoresis does not come with that luxury and often requires multiple attempts [101].

Recently, different attempts were carried out by researchers to improve the sensitivity of the LAMP-LFAs. One of those was to introduce filtration-based LAMP-LFA. In this assay, the microbial cells were concentrated by filtration instead of cell enrichment culture. A cellulose acetate filter was used to concentrate the *Escherichia coli* cells in this research, improving the concentration of the sample required for analysis. The genomic samples were then isothermally amplified using LAMP and visually detected by LFA. The outcome was overwhelming, but the overall process took a long time and does not suit the criteria of POCT devices [60]. In another research, LAMP-LFAs are further modified into multiplex LFA to use them for the detection of more than one sample at a time. A bacterium that has heat-stable protein resistant to protease in the gastrointestinal tract that can cause food poisoning is observed through this format. Multiplex LAMP-LFAs help to carry out multiple tests at one time using a single membrane, hence greatly reducing the need for repetitive LAMP and a sample application with different LFA kits [61]. Recently, an RT-LAMP assay was developed to detect SARS-CoV-2 in real-time without using an RNA extraction kit. The test was fast and effective and believed to be one of the alternatives to detect the presence of the virus at the patient's site without analyzing in centralized laboratories [99, 100].

## Aptamer–based NALFAs

Antibodies have been and are still being used as a key recognition element in assays. Monoclonal antibodies such as mouse hybridomas are widely used nowadays due to their proven affinity and specificity [46]. However, denaturation induced by temperature, time-consuming and laborious chemical modifications, and not suitable for nonimmunogenic detection makes the antibodies a vulnerable choice to be used in LFA without further modifications. As an alternative, aptamers became a preferred choice for the detection strategy due to their advantage over the areas where antibodies lack [102].

First introduced in the 1990s, aptamers are short single-stranded DNA or RNA sequences. They possess enormous binding affinity and sensitivity for molecular targets ranging from macromolecules to micromolecules [89]. Unlike antibodies, aptamers have no batch-to-batch variation, have extended shelf-life with very minimal immunogenicity, and easily get along with any chemical modifications to improve the binding capacity. Due to these advantages, aptamers were incorporated in LFAs replacing antibodies, and different types of aptamers-based LFAs (Apt-LFAs) were subjected for use in various fields such as disease diagnosis, agriculture, and food industry. Despite that, these Apt-LFAs are not commercially available yet and still fall behind antibody-based LFAs in the aspect of general use [102].

Aptamers and antibodies have similarities in terms of tertiary structure-based target recognition [89]. Hence, most Apt-LFAs are designed based on the idea of antibody-based LFAs. They are abundantly used for the sandwich assay method [103] compared to the competitive assay method due to their ability to easily recognize large molecules such as proteins. The first sandwich Apt-LFA was developed for thrombin detection using dual aptamers that can target different recognition sites of thrombin molecules. The first aptamer was conjugated to the AuNPs and loaded onto the conjugate pad, serving as reporter aptamer. The reporter aptamer also includes a short poly-A tail, which later is easily recognized by the complementary sequence with an additional poly-T tail at the control line. Then, the biotinylated second aptamer immobilized onto the test zone via streptavidin-biotin interaction, serving as capture aptamer. Both the aptamers were able to bind to different sides of the thrombin, forming a sandwich complex and eventually producing a red signal at the test zone. A red signal was also produced at the control zone because of the interaction between the unbound detection aptamer conjugate and oligonucleotide sequences at the control zone that is a complementary detection aptamer. This dual aptamer strategy was highly preferred due to its high sensitivity and unmatched success rate. In recent years, aptamer–antibody combination and split aptamer strategies were used but their applications later were ceased due to instability and low sensitivity [102].

For the competitive assay, dual aptamers are not used because naturally this type of assay is performed for smaller molecules with fewer recognition sites. In this assay, an oligonucleotide partially complementary to the aptamer sequence will be immobilized onto the test line. When target analyte is applied onto the sample pad, they migrate and eventually are bound to the aptamer–reporter complex. When the newly formed complex reaches the test line, a competition takes place between the targets in the sample and the preloaded targets upon the binding affinity toward the detection aptamer, and no signal will be produced at the test zone. If target analytes are absent in the sample, then the aptamer–reporter complex is easily detected by the preloaded target at the test line, forming a strong signal. The interaction between detection aptamer and the oligonucleotide sequence complementary to detection aptamer immobilized at control line produces a signal, validating the LFA [91].

Aptamer immobilization onto the membranes is an important part of the Apt-LFA fabrication [89]. Usually, noncovalent strategies are used to immobilize the aptamers onto the nitrocellulose membrane. Biotin and streptavidin are the important and most-used components for noncovalent attachment. The biotin molecules will be conjugated at the end of the aptamer and later incubated with membrane-immobilized streptavidin to form a strong binding between the aptamer and the membrane with high affinity [104]. The use of biotin–streptavidin interaction often results in stability issues, making them incompatible with nucleic acid aptamers. Hence, microgel-mediated immobilization, tandem repeating–mediated aptamer immobilization, and ultraviolet (UV)–based covalent binding immobilization strategies are now progressively tested for strong immobilization [102].

## Conclusion

The POCT has completely shifted the paradigm of diagnosis strategy in today's world. The development of LFAs with greater sensitivity and specificity, while they emerged as highly cost-effective technologies, enabled fast diagnosis of many infectious diseases. At the same time, LFAs that use nucleic acid samples for detection such as NALFAs improve the detection sensitivity and duration for NCDs, further expanding the administration of POCT in various fields to replace laborious conventional methods.

Despite these advantages, the requirement for confirmatory independent tests for certain conditions, the increase in cost for qualitative analysis, and technical blunders are a few shortcomings often associated with POCT devices, particularly for LFAs. However, the recent and prospective innovations targeted upon POCT and LFA can improve the effectiveness of this technology and make it indispensable for the future.

## Abbreviations

ABICAP: Antibody Immuno Column for Analytical Processes;
Apt-LFA: Aptamer-based LFA;
ARMS-LFA: amplification refractory mutation system-LFA;
ASSURED: affordable, sensitive and specific, user-friendly, rapid and robust equipment-free and deliverable;
ATP: adenosine triphosphate;
AuNPs: Gold nanoparticles;
Bst: *Bacillus stearothermophilus*;
CNTs: carbon nanotubes;
*E. coli*: *Escherichia coli*;
HAD: helicase-dependent amplification;
HAT: hemagglutination test;
HIV: human immunodeficiency virus;
HPLC: high-performance liquid chromatography;
HRP: Horseradish peroxidase;
ICS: immunochromatographic strip;
INR: international normalized ratio;
LAMP: loop-mediated isothermal amplification;
LFAs: lateral flow assays;
LFDs: lateral flow devices;
LOD: limit of detection;
MERS-CoV: Middle East respiratory syndrome-based coronavirus;
MNPs: magnetic nanoparticles;
MPQ: magnetic particles quantification;
NABs: nucleic acid biosensors;
NALFAs: nucleic acid lateral flow assays;
NALFIA: nucleic acid lateral flow immunoassay;
NASBA: nucleic acid sequence-based amplification;
NGS: next-generation sequencing;
NUS: National University of Singapore;
PA: photo acoustic;
PCR: polymerase chain reaction;
POC: point-of-care;
POCT: point-of-care testing;
PT: photothermal;
RBD: receptor binding domain;
RCA: rolling circle amplification;
RPA: recombinase polymerase amplification;
RT-PCR: reverse transcription-polymerase chain reaction;
RT-qPCR: reverse transcription-quantitative polymerase chain reaction;
SARS-CoV-2: severe acute respiratory syndrome coronavirus 2;
SERS: surface-enhanced Raman scattering;
SPR: surface plasmon resonance;
UV: ultraviolet;
WHO: World Health Organization.

## References

[1] Yager P, Domingo GJ, Gerdes J. (2008). Point-of-care diagnostics for global health. Annu Rev Biomed Eng..

[2] Ahsan W, Alhazmi HA, Patel KS, Mangla B, Al Bratty M, Javed S (2020). Recent advancements in the diagnosis, prevention, and prospective drug therapy of COVID-19. Front Public Heal..

[3] Bunn TW, Sikarwar A. (2016). Diagnostics: conventional versus modern methods. J Adv Med Pharm Sci..

[4] Franco-Duarte R, Černáková L, Kadam S, Kaushik KS, Salehi B, Bevilacqua A (2019). Advances in chemical and biological methods to identify microorganisms—from past to present. Microorganisms..

[5] Weile J, Knabbe C. (2009). Current applications and future trends of molecular diagnostics in clinical bacteriology. Anal Bioanal Chem..

[6] Leland DS, Ginocchio CC. (2007). Role of cell culture for virus detection in the age of technology. Clin Microbiol Rev..

[7] Warsinke A. (2009). Point-of-care testing of proteins. Anal Bioanal Chem..

[8] Patrinos GP, Danielson PB, Ansorge WJ. (2017). Molecular diagnostics: past, present, and future.

[9] Raghavendra P, Pullaiah T. (2018). Future of cellular and molecular diagnostics.

[10] Choi JR, Yong KW, Choi JY, Cowie AC. (2019). Emerging point-of-care technologies for food safety analysis. Sensors (Basel, Switzerland)..

[11] Charlermroj R, Phuengwas S, Makornwattana M, Sooksimuang T, Sahasithiwat S, Panchan W (2021). Development of a microarray lateral flow strip test using a luminescent organic compound for multiplex detection of five mycotoxins. Talanta..

[12] Yager P, Edwards T, Fu E, Helton K, Nelson K, Tam MR, Weigl BH. (2006). Microfluidic diagnostic technologies for global public health. Nature..

[13] Bouzid D, Zanella MC, Kerneis S, Visseaux B, May L, Schrenzel J, Cattoir V. (2021). Rapid diagnostic tests for infectious diseases in the emergency department. Clin Microbiol Infect..

[14] Napione L. (2021). Integrated nanomaterials and nanotechnologies in lateral flow tests for personalized medicine applications. Nanomaterials (Basel)..

[15] Von Lode P. (2005). Point-of-care immunotesting: approaching the analytical performance of central laboratory methods. Clin Biochem..

[16] Carter DJ, Cary RB. (2007). Lateral flow microarrays: a novel platform for rapid nucleic acid detection based on miniaturized lateral flow chromatography. Nucleic Acids Res..

[17] Yetisen AK, Akram MS, Lowe CR. (2013). Paper-based microfluidic point-of-care diagnostic devices. Lab Chip..

[18] John AS, Price CP. (2014). Existing and emerging technologies for point-of-care testing. Clin Biochem Rev..

[19] Kost GJ. (2019). Geospatial science and point-of-care testing: creating solutions for population access, emergencies, outbreaks, and disasters. Front Pub Health..

[20] Chen H, Liu K, Li Z, Wang P. (2019). Point of care testing for infectious diseases. Clin Chim Acta..

[21] Kim S, Nhem S, Dourng D, Ménard D. (2015). Malaria rapid diagnostic test as point-of-care test: study protocol for evaluating the VIKIA® Malaria Ag Pf/Pan. Malar J..

[22] Lee WG, Kim YG, Chung BG, Demirci U, Khademhosseini A. (2010). Nano/Microfluidics for diagnosis of infectious diseases in developing countries. Adv Drug Deliv Rev..

[23] Qiu X, Sokoll L, Yip P, Elliott DJ, Dua R, Mohr P (2017). Comparative evaluation of three FDA-approved HIV Ag/Ab combination tests using a genetically diverse HIV panel and diagnostic specimens. J Clin Virol..

[24] Campos NG, Tsu V, Jeronimo J, Mvundura M, Kim JJ. (2017). Estimating the value of point-of-care HPV testing in three low- and middle-income countries: a modeling study. BMC Cancer..

[25] Broadhurst MJ, Kelly JD, Miller A, Semper A, Bailey D, Groppelli E (2015). ReEBOV antigen rapid test kit for point-of-care and laboratory-based testing for Ebola virus disease: a field validation study. Lancet..

[26] Maniki PT, Khan R, Orchard A, De Rapper S, Padayachee N. (2022). Promoting the use of point of care testing in non-communicable disease screening among university students. Int J Africa Nurs Sci..

[27] Gbinigie O, Price CP, Heneghan C, Van den Bruel A, Plüddemann A. (2015). Creatinine point-of-care testing for detection and monitoring of chronic kidney disease: primary care diagnostic technology update. Br J Gen Pract..

[28] Beaney T, Burrell LM, Castillo RR, Charchar FJ, Cro S, Damasceno A (2019). May measurement month 2018: a pragmatic global screening campaign to raise awareness of blood pressure by the international society of hypertension. Eur Heart J..

[29] Hayes B, Murphy C, Crawley A, O’Kennedy R. (2018). Developments in point-of-care diagnostic technology for cancer detection. Diagnostics (Basel)..

[30] Kristensen T, Rose-Olsen K, Volmar Skovsgaard C. (2020). Effects of point-of-care testing in general practice for type 2 diabetes patients on ambulatory visits and hospitalizations. Int J Environ Res Public Health..

[31] Valera E, Jankelow A, Lim J, Kindratenko V, Ganguli A, White K (2021). COVID-19 point-of-care diagnostics: present and future. ACS Nano..

[32] Kierkegaard P, McLister A, Buckle P. (2021). Rapid point-of-care testing for COVID-19: quality of supportive information for lateral flow serology assays. BMJ Open..

[33] Nilghaz A, Wicaksono DH, Gustiono D, Abdul Majid FA, Supriyanto E, Abdul Kadir MR. (2012). Flexible microfluidic cloth-based analytical devices using a low-cost wax patterning technique. Lab Chip..

[34] Hu J, Wang S, Wang L, Li F, Pingguan-Murphy B, Lu TJ, Xu F. (2014). Advances in paper-based point-of-care diagnostics. Biosens Bioelectron..

[35] Sharma S, Zapatero-Rodríguez J, Estrela P, O’Kennedy R. (2015). Point-of-care diagnostics in low resource settings: present status and future role of microfluidics. Biosensors (Basel)..

[36] Luppa PB, Müller C, Schlichtiger A, Schlebusch H. (2011). Point-of-care testing (POCT): current techniques and future perspectives. Trends Analyt Chem..

[37] Townsend A, Rijal P, Xiao J, Tan TK, Huang KA, Schimanski L (2021). A haemagglutination test for rapid detection of antibodies to SARS-CoV-2. Nat Commun..

[38] Wood JR, Kaminski BM, Kollman C, Beck RW, Hall CA, Yun JP (2012). Accuracy and precision of the axis-shield afinion hemoglobin A1c measurement device. J Diabetes Sci Technol..

[39] Stern D, Olson VA, Smith SK, Pietraszczyk M, Miller L, Miethe P (2016). Rapid and sensitive point-of-care detection of Orthopox-viruses by ABICAP immunofiltration. Virol J..

[40] Jiang X, Lillehoj PB. (2021). Lateral flow immunochromatographic assay on a single piece of paper. Analyst..

[41] Prasad S. (2014). Nanobiosensors: the future for diagnosis of disease?. Nanobiosensors Dis Diagn..

[42] Solaimuthu A, Vijayan AN, Murali P, Korrapati PS. (2020). Nano-biosensors and their relevance in tissue engineering. Curr Opin Biomed Eng..

[43] Abdel-Karim R, Reda Y, Abdel-Fattah A. (2020). Review—nanostructured materials-based nanosensors. J Electrochem Soc..

[44] Noah NM, Ndangili PM. (2019). Current trends of nanobiosensors for point-of-care diagnostics. J Anal Methods Chem..

[45] Antiochia R. (2020). Nanobiosensors as new diagnostic tools for SARS, MERS and COVID-19: from past to perspectives. Mikrochim Acta..

[46] Koczula KM, Gallotta A. (2016). Lateral flow assays. Essays Biochem..

[47] Posthuma-Trumpie GA, Korf J, van Amerongen A. (2009). Lateral flow (immuno) assay: its strengths, weaknesses, opportunities and threats. a literature survey. Anal Bioanal Chem..

[48] Posthuma-Trumpie GA, van Amerongen A. (2012). Lateral flow assays. Antibodies Appl New Dev..

[49] Sajid M, Kawde AN, Daud M. (2015). Designs, formats and applications of lateral flow assay: a literature review. J Saudi Chem Soc..

[50] Edwards KA, Baeumner AJ. (2006). Optimization of DNA-tagged dye-encapsulating liposomes for lateral-flow assays based on sandwich hybridization. Anal Bioanal Chem..

[51] Yoo EH, Lee SY. (2010). Glucose biosensors: an overview of use in clinical practice. Sensors (Basel)..

[52] Paek SH, Lee SH, Cho JH, Kim YS. (2000). Development of rapid one-step immunochromatographic assay. Methods..

[53] Nguyen VT, Song S, Park S, Joo C. (2020). Recent advances in high-sensitivity detection methods for paper-based lateral-flow assay. Biosens Bioelectron..

[54] Wang D, He S, Wang X, Yan Y, Liu J, Wu S (2020). Rapid lateral flow immunoassay for the fluorescence detection of SARS-CoV-2 RNA. Nat Biomed Eng..

[55] Khlebtsov B, Khlebtsov N. (2020). Surface-enhanced Raman scattering-based lateral-flow immunoassay. Nanomaterials (Basel)..

[56] Calabria D, Calabretta MM, Zangheri M, Marchegiani E, Trozzi I, Guardigli M (2021). Recent advancements in enzyme-based lateral flow immunoassays. Sensors (Basel)..

[57] Qu Z, Wang K, Alfranca G, de la Fuente JM, Cui D. (2020). A plasmonic thermal sensing based portable device for lateral flow assay detection and quantification. Nanoscale Res Lett..

[58] Ye H, Liu Y, Zhan L, Liu Y, Qin Z. (2020). Signal amplification and quantification on lateral flow assays by laser excitation of plasmonic nanomaterials. Theranostics..

[59] Resch-Genger U, Barker PE. (2008). Standardization and quality assurance in fluorescence measurements. II Bioanalytical and biomedical applications..

[60] Henderson WA, Xiang L, Fourie NH, Abey SK, Ferguson EG, Diallo AF (2018). Simple lateral flow assays for microbial detection in stool. Anal Methods..

[61] Yin HY, Fang TJ, Wen HW. (2016). Combined multiplex loop-mediated isothermal amplification with lateral flow assay to detect sea and *seb* genes of enterotoxic *Staphylococcus aureus*. Lett Appl Microbiol..

[62] O’Farrell B., Wong R, Tse H (2009). Evolution in lateral flow – based immunoassay systems. Lateral flow immunoassay.

[63] Ching KH. (2015). Lateral flow immunoassay. Methods Mol Biol..

[64] de Assis TSM, Freire ML, Carvalho JP, Rabello A, Cota G. (2022). Cost-effectiveness of anti-SARS-CoV-2 antibody diagnostic tests in Brazil. PLoS One..

[65] Ramachandran A, Manabe Y, Rajasingham R, Shah M. (2017). Cost-effectiveness of CRAG-LFA screening for cryptococcal meningitis among people living with HIV in Uganda. BMC Infect Dis..

[66] Goldstein LN, Wells M, Vincent-Lambert C. (2019). The cost-effectiveness of upfront point-of-care testing in the emergency department: a secondary analysis of a randomised, controlled trial. Scand J Trauma Resusc Emerg Med..

[67] Harder R, Wei K, Vaze V, Stahl JE. (2019). Simulation analysis and comparison of point of care testing and central laboratory testing. MDM Policy Pract..

[68] Osredkar J. (2017). Point-of-care testing in laboratory medicine. Point-of-care diagnostics - new progresses perspectives..

[69] Lin M, Zhao Y, Wang S, Liu M, Duan Z, Chen Y (2012). Recent advances in synthesis and surface modification of lanthanide-doped upconversion nanoparticles for biomedical applications. Biotechnol Adv..

[70] Liu L, Yang D, Liu G. (2019). Signal amplification strategies for paper-based analytical devices. Biosens Bioelectron..

[71] Andryukov BG. (2020). Six decades of lateral flow immunoassay: from determining metabolic markers to diagnosing covid-19. AIMS Microbiol..

[72] Guteneva NV, Znoyko SL, Orlov AV, Nikitin MP, Nikitin PI. (2019). Rapid lateral flow assays based on the quantification of magnetic nanoparticle labels for multiplexed immunodetection of small molecules: application to the determination of drugs of abuse. Microchim Acta..

[73] Zhang Y, Liu X, Wang L, Yang H, Zhang X, Zhu C (2020). Improvement in detection limit for lateral flow assay of biomacro-molecules by test-zone pre-enrichment. Sci Rep..

[74] Kapoor A, Balasubramanian S, Vaishampayan V, Ghosh R (2018). Lab-on-a-chip: a potential tool for enhancing teaching-learning in developing countries using paper microfluidics. Int Conf Transform Eng Educ ICTEE 2017.

[75] Di Nardo F, Chiarello M, Cavalera S, Baggiani C, Anfossi L. (2021). Ten years of lateral flow immunoassay technique applications: trends, challenges and future perspectives. Sensors (Basel)..

[76] Bartosh AV, Sotnikov DV, Hendrickson OD, Zherdev AV, Dzantiev BB. (2020). Design of multiplex lateral flow tests: a case study for simultaneous detection of three antibiotics. Biosensors (Basel)..

[77] Anfossi L, Di Nardo F, Cavalera S, Giovannoli C, Baggiani C. (2018). Multiplex lateral flow immunoassay: an overview of strategies towards high-throughput point-of-need testing. Biosensors (Basel)..

[78] Omidfar K, Khorsand F, Darziani Azizi M. (2013). New analytical applications of gold nanoparticles as label in antibody based sensors. Biosens Bioelectron..

[79] Pongsuchart M, Sereemaspun A, Ruxrungtham K. (2013). UV treatment nucleic acid probe without biotin-labeling is sensitive and sufficient for the fabrication of nucleic acid lateral flow (NALF) strip test. J Life Sci Technol..

[80] Pecchia S, Da Lio D. (2018). Development of a rapid PCR-nucleic acid lateral flow immunoassay (PCR-NALFIA) based on rDNA IGS sequence analysis for the detection of macrophomina phaseolina in soil. J Microbiol Methods..

[81] Liu X, Zhang C, Liu K, Wang H, Lu C, Li H (2018). Multiple SNPs detection based on lateral flow assay for phenylketonuria diagnostic. Anal Chem..

[82] Stedtfeld RD, Tourlousse DM, Seyrig G, Stedtfeld TM, Kronlein M, Price S (2012). Gene-Z: a device for point of care genetic testing using a smartphone. Lab Chip..

[83] Ho NRY, Lim GS, Sundah NR, Lim D, Loh TP, Shao H. (2018). Visual and modular detection of pathogen nucleic acids with enzyme–DNA molecular complexes. Nat Commun..

[84] Ivanov AV, Safenkova IV, Zherdev AV, Dzantiev BB. (2020). Nucleic acid lateral flow assay with recombinase polymerase amplification: solutions for highly sensitive detection of RNA virus. Talanta..

[85] Sabidó M, Hernandez G, Gonzalez V, Valles X, Montoliu A, Figuerola J (2009). Clinic-based evaluation of a rapid point-of-care test for detection of Chlamydia trachomatis in specimens from sex workers in Escuintla, Guatemala. J Clin Microbiol..

[86] Krõlov K, Frolova J, Tudoran O, Suhorutsenko J, Lehto T, Sibul H (2014). Sensitive and rapid detection of chlamydia trachomatis by recombinase polymerase amplification directly from urine samples. J Mol Diagnostics..

[87] Mens PF, de Bes HM, Sondo P, Laochan N, Keereecharoen L, van Amerongen A (2012). Direct blood PCR in combination with nucleic acid lateral flow immunoassay for detection of Plasmodium species in settings where malaria is endemic. J Clin Microbiol..

[88] Varlamov DA, Blagodatskikh KA, Smirnova EV, Kramarov VM, Ignatov KB. (2020). Combinations of PCR and isothermal amplification techniques are suitable for fast and sensitive detection of SARS-CoV-2 viral RNA. Front Bioeng Biotechnol..

[89] Li F, Zhang H, Wang Z, Newbigging AM, Reid MS, Li XF, Le XC. (2015). Aptamers facilitating amplified detection of biomolecules. Anal Chem..

[90] Javani A, Javadi-Zarnaghi F, Rasaee MJ. (2017). Development of a colorimetric nucleic acid-based lateral flow assay with non-biotinylated capture DNA. Appl Biol Chem..

[91] Jauset-Rubio M, Svobodova M, Mairal T, McNeil C, Keegan N, Saeed A (2016). Ultrasensitive, rapid and inexpensive detection of DNA using paper based lateral flow assay. Sci Rep..

[92] Liu Y, Zhan L, Qin Z, Sackrison J, Bischof JC. (2021). Ultrasensitive and highly specific lateral flow assays for point-of-care diagnosis. ACS Nano..

[93] Crannell ZA, Rohrman B, Richards-Kortum R. (2014). Equipment-free incubation of recombinase polymerase amplification reactions using body heat. PLoS One..

[94] Mugasa CM, Laurent T, Schoone GJ, Kager PA, Lubega GW, Schallig HD. (2009). Nucleic acid sequence-based amplification with oligochromatography for detection of trypanosoma brucei in clinical samples. J Clin Microbiol..

[95] Wu Q, Suo C, Brown T, Wang T, Teichmann SA, Bassett AR. (2021). INSIGHT: a population-scale COVID-19 testing strategy combining point-of-care diagnosis with centralized high-throughput sequencing. Sci Adv..

[96] Hara-Kudo Y, Konishi N, Ohtsuka K, Hiramatsu R, Tanaka H, Konuma H, Takatori K. (2008). Detection of verotoxigenic *Escherichia coli* O157 and O26 in food by plating methods and LAMP method: a collaborative study. Int J Food Microbiol..

[97] Nizar NNA, Zainal IH, Bonny SQ, Pulingam T, Vythalingam LM, Ali ME. (2018). DNA and nanobiosensor technology for the detection of adulteration and microbial contamination in religious food.

[98] Foo PC, Nurul Najian AB, Muhamad NA, Ahamad M, Mohamed M, Yean Yean C, Lim BH. (2020). Loop-mediated isothermal amplification (LAMP) reaction as viable PCR substitute for diagnostic applications: a comparative analysis study of LAMP, conventional PCR, nested PCR (nPCR) and real-time PCR (qPCR) based on Entamoeba histolytica DNA derived from. BMC Biotechnol..

[99] Hardinge P, Murray JAH. (2019). Reduced false positives and improved reporting of loop-mediated isothermal amplification using quenched fluorescent primers. Sci Rep..

[100] Zhao X, Lin CW, Wang J, Oh DH. (2014). Advances in rapid detection methods for foodborne pathogens. J Microbiol Biotechnol..

[101] Panno S, Matić S, Tiberini A, Caruso AG, Bella P, Torta L (2020). Loop mediated isothermal amplification: principles and applications in plant virology. Plants (Basel)..

[102] Wang T, Chen L, Chikkanna A, Chen S, Brusius I, Sbuh N, Veedu RN. (2021). Development of nucleic acid aptamer-based lateral flow assays: a robust platform for cost-effective point-of-care diagnosis. Theranostics..

[103] Liu G, Mao X, Phillips JA, Xu H, Tan W, Zeng L. (2009). Aptamer-nanoparticle strip biosensor for sensitive detection of cancer cells. Anal Chem..

[104] Zhou L, Wang MH, Wang JP, Ye ZZ. (2011). Application of biosensor surface immobilization methods for aptamer. Chin J Anal Chem..

